# Annual acknowledgement of manuscript reviewers

**DOI:** 10.1186/1746-4269-9-16

**Published:** 2013-03-21

**Authors:** Andrea Pieroni

**Affiliations:** 1 University of Gastronomic Sciences, Piazza Vittorio Emanuele 9, 12060 Bra/Pollenzo, Cuneo, Italy

## Abstract

**Contributing reviewers:**

The editors of Journal of Ethnobiology and Ethnomedicine would like to thank all our reviewers who have contributed to the journal in Volume 8 (2012).

## 

Raziq Abdul

Pakistan

Talal Aburjai

Jordan

Ulysses P. de Albuquerque

Brazil

Mohammed Ali-Shtayeh

Palestinian Territory

Romulo Alves

Brazil

E. N. Anderson

United States of America

Fabiana Antognoni

Ireland

Zemede Asfaw

Ethiopia

Muniappan Ayyanar

India

Alpina Begossi

Brazil

Shandesh Bhattarai

Nepal

Eric Boa

United Kingdom

Roel Bosma

Netherlands

Carole Browner

United States of America

Piero Bruschi

Italy

Alejandro Casas

Mexico

Michael J. Casimir

Germany

Victoria Castro

Chile

Luis Ceríaco

Portugal

Melissa Ceuterick

United Kingdom

Angelo Giuseppe Chaves Alves

Brazil

Xiaoming Chen

China

Anthony Davis

Canada

Hugo de Boer

Sweden

Daniele De Meneghi

Italy

W. Bernhard Dickoré

Germany

Bethany Elkington

United States of America

Fusun Ertug

Turkey

Bernard Faye

Saudi Arabia

Felipe Ferreira

Brazil

Maria Franco T. Medeiros

Brazil

Jo Frencken

Netherlands

R. T. Gahukar

India

Adebayo Gbolade

Nigeria

Abdolbaset Ghorbani

Iran

Mirutse Giday

Ethiopia

Peter Giovannini

United Kingdom

M. Reyes González-Tejero

Spain

Andrew Gosler

United Kingdom

Maximilien Gueze

Spain

Simon Haberle

Australia

Natalia Hanazaki

Brazil

M. Diane Hardgrave

United States of America

Eugene Hunn

United States of America

Savarimuthu Ignacimuthu

India

Abdul Jabbar

Australia

Anita Jain

India

Kurt Jax

Germany

Hannah Jennings

United Kingdom

Kevin Jernigan

United States of America

Leslie Main Johnson

Canada

Ripu Kunwar

Nepal

Ana Haydee Ladio

Argentina

Stacey Langwick

United States of America

Cheryl Lans

United Kingdom

Marco Leonti

Italy

Efraim Lev

Israel

Chunlin Long

China

Alan Loures-Ribeiro

Brazil

Lukasz Luczaj

Poland

Ermias Lulekal

Ethiopia

Manuel J. Macia

Spain

Alfred Maroyi

South Africa

Patrick Masika

South Africa

Evelyn Mathias

Germany

Francesc Maynou

Spain

Heather McMillen

United States of America

Brien Meilleur

France

Victor Benno Meyer-Rochow

Germany

Javier Mignone

Canada

Elizabeth Miller

United States of America

Pozi Milow

Malaysia

Helder Neves de Albuquerque

Brazil

Adolphe Nfotabong-Atheull

Belgium

Justin Nolan

United States of America

Nivaldo Nordi

Brazil

Olawole Obembe

Nigeria

Nkechi Offiah

Trinidad and Tobago

Manuel Pardo-de-Santayana

Spain

Mariève Pouliot

Denmark

Tom Prescott

United Kingdom

Lisa Leimar Price

Netherlands

Cassandra Quave

United States of America

Marsha Quinlan

United States of America

Rahmatullah Qureshi

Pakistan

Subramanyam Ragupathy

Canada

Mohammed Rahmatullah

Bangladesh

Julieta Ramos-Elorduy

Mexico

Sulejman Redzic

Bosnia and Herzegovina

Victoria Reyes-Garcia

Spain

Eliana Rodrigues

Brazil

Tonjock Rosemary Kinge

Cameroon

Ricardo Rozzi

United States of America

Roger Sansi

United States of America

Valentina Savo

Italy

Gary D. Schnell

United States of America

Krishna Shrestha

Nepal

Maria Adele Signorini

Italy

Renato Silvano

Brazil

Derek Smith

Canada

Renata Soukand

Estonia

Torunn Stangeland

Norway

Rick Stepp

United States of America

Ingvar Svanberg

Sweden

Olivia Sylvester

Canada

John R. S. Tabuti

Uganda

Javier Tardio

Spain

Juergen Tautz

Germany

Evert Thomas

Belgium

Thomas Tobin

United States of America

Will Tuladhar-Douglas

Nepal

Joan Vallès

Spain

Tinde van Andel

Netherlands

Sjaak van der Geest

Netherlands

Arnold van Huis

Netherlands

Ina Vandebroek

United States of America

Alfredo Vannacci

Italy

Giuseppe Venturella

Italy

Christian R. Vogl

Austria

Martin Walsh

United Kingdom

Stanley Wambugu

Kenya

Caroline Weckerle

Switzerland

Mary S. Willis

United States of America

Felice Wyndham

Canada

Jaya Parkash Yadav

India

Alan Yen

Australia

Erdem Yesilada

Turkey

Haile Yineger

Ethiopia

Zhongzhen Zhao

Hong Kong

